# Epitope mapping of an anti-diacylglycerol kinase delta monoclonal antibody DdMab-1

**DOI:** 10.1016/j.bbrep.2020.100808

**Published:** 2020-09-02

**Authors:** Masato Sano, Teizo Asano, Mika K. Kaneko, Yukinari Kato

**Affiliations:** a Department of Antibody Drug Development, Tohoku University Graduate School of Medicine, 2-1 Seiryo-machi, Aoba-ku, Sendai, Miyagi, 980-8575, Japan; b New Industry Creation Hatchery Center, Tohoku University, 2-1, Seiryo-machi, Aoba-ku, Sendai, Miyagi, 980-8575, Japan

**Keywords:** DGKd, DdMab-1, Monoclonal antibody, DGKδ, Diacylglycerol kinase δ, hDGKδ, human DGKδ, mAb, monoclonal antibody, PBS, phosphate-buffered saline, PEG, polyethylene glycol, PH, pleckstrin homology, PVDF, polyvinylidene difluoride, SAM, sterile alpha motif, TBS, Tris-buffered saline

## Abstract

Diacylglycerol kinase δ (DGKδ) is a type II DGK, which catalyzes diacylglycerol phosphorylation to produce phosphatidic acid. DGKδ is expressed in several types of tissues and organs including the stomach, testis, bone marrow, and lymph node. Here, we established an anti-human DGKδ (hDGKδ) mAb, DdMab-1 (mouse IgG_2a_, kappa), which is useful for Western blot analysis. We also introduced deletion or point mutations to hDGKδ, and performed western blotting to determine the binding epitope of DdMab-1. DdMab-1 reacted with the dN670 mutant, but not with the dN680 mutant, indicating that the N-terminus of the DdMab-1 epitope is mainly located between amino acids 670 and 680 of the protein. Further analysis using point mutants demonstrated that R675A, R678A, K679A, and K682A mutants were not detected, and V680A was only weakly detected by DdMab-1, indicating that Arg675, Arg678, Lys679, Val680 and Lys682 are important for binding of DdMab-1 to hDGKδ.

## Highlights

• DGKδ catalyzes diacylglycerol phosphorylation to produce phosphatidic acid.
• We established a novel anti-hDGKδ mAb, DdMab-1.
• DdMab-1 is useful for Western blot analysis.
• R675, R678, K679, V680, and K682 are important for binding of DdMab-1 to hDGKδ.

## 1 Introduction

Diacylglycerol kinase (DGK) plays a critical role in the regulation of numerous cellular functions by catalyzing the phosphorylation of diacylglycerol to phosphatidic acid [1,2]. Diacylglycerol activates protein kinase C, and the DGK terminates the diacylglycerol-mediated signaling pathway by phosphorylating diacylglycerol [[3], [4], [5], [6], [7]]. Here, the resulting phosphatidic acid functions as a second messenger which regulates the intracellular Ca^2+^ level and the mTOR-mediated signaling pathway [8,9].

Ten isozymes of the DGK family have been so far identified in mammals [2]. DGK family is also grouped into five subtypes based on their subtype-specific functional domains. DGKδ is one of the DGK family, and was first cloned from the human testis cDNA library [10].

DGKδ is expressed in several tissues and organs including the stomach, testis, bone marrow, and lymph node [11]. DGKδ is a type II DGK which contains pleckstrin homology (PH) and sterile alpha motif (SAM) domains at the N- and C-terminus of the protein, respectively. The PH domain can bind protein kinase C, the βγ-subunits of heterotrimeric G proteins, and phosphatidylinositol 4,5-bisphosphate [[12], [13], [14]]. On the other hand, the SAM domain has been shown to mediate both homo- and hetero-oligomerization, and therefore is a putative protein interaction module [15,16].

DGKδ was previously shown to regulate protein kinase C activity, and thereby control the degradation of epidermal growth factor receptor via modulation of ubiquitin-specific protease 8 expression in cultured human cells [17,18]. Moreover, DGKδ expression and activity levels are reduced in skeletal muscle tissues of Type 2 diabetic patients [19]. Hence, an anti-DGKδ monoclonal antibody (mAb) is required for specific detection of DGKδ in human tissues.

In this study, we established a novel anti-human DGKδ (hDGKδ) mAb, DdMab-1, by immunizing mice with recombinant hDGKδ. We also determined the binding epitope of DdMab-1 using deletion or point mutants of hDGKδ via Western blot analysis.

## 2 Materials and methods

### 2.1 Plasmid preparation

Synthesized DNA (Eurofins Genomics KK, Tokyo, Japan) encoding hDGKδ (accession No.NM_152879) plus a C-terminal PA tag (GVAMPGAEDDVV) [20] was subcloned into the pMAL-c2 expression vector (New England Biolabs Inc., Beverly, MA) using the In-Fusion HD Cloning Kit (Takara Bio, Inc., Shiga, Japan). The PA tag is recognized by an anti-PA tag mAb (NZ-1) [21]. The resulting construct was named pMAL-c2-hDGKδ-PA. The deletion mutants of hDGKδ DNA were amplified via polymerase chain reaction, and subcloned into the pMAL-c2 with a PA tag using the In-Fusion HD Cloning Kit. The substitution of hDGKδ amino acids with alanine on dN610 of hDGKδ was performed using the QuikChange Lightning Site-Directed Mutagenesis Kit (Agilent Technologies, Inc., Santa Clara, CA, USA). These constructs were also verified by direct DNA sequencing.

### 2.2 Production of the recombinant DGKδ protein

Competent *E. coli* TOP-10 cells (Thermo Fisher Scientific Inc., Waltham, MA, USA) were transformed with the pMAL-c2-hDGKδ-PA plasmid. The cells were cultured overnight at 37 °C in LB medium (Thermo Fisher Scientific Inc.) containing 100 μg/ml of ampicillin (Sigma-Aldrich Corp., St. Louis, MO). Cell pellets were resuspended in phosphate-buffered saline (PBS) containing 1% Triton X-100 and 50 μg/ml aprotinin (Sigma-Aldrich Corp.). After sonication, crude extracts were collected using centrifugation (9000×*g*, 30 min, 4 °C). The lysates were passed through a 0.45 μm filter to remove trace amounts of insoluble materials. Filtered lysates were then mixed with NZ-1-Sepharose (1 ml of bed volume), and incubated at 4 °C for 2 h under gentle agitation. The resulting resin was then transferred to a column, and washed with 20 ml of Tris-buffered saline (TBS; pH 7.5). The bound protein was eluted with the PA tag peptide at room temperature in a stepwise manner (1 ml × 10 washes).

### 2.3 Hybridoma production

The Animal Care and Use Committee of Tohoku University approved all animal experiments. DdMab-1 was produced using the mouse medial iliac lymph node method. Briefly, three female 8-week old B6D2F1/Slc mice (Japan SLC Inc., Shizuoka, Japan) were immunized by injecting 33 μg of the pMAL-c2-hDGKδ-PA protein and Freund's complete adjuvant (Sigma-Aldrich Corp.) into their footpad. Additional immunization with 50 μg of the pMAL-c2-hDGKδ-PA protein was performed via the tail base. The lymphocytes were fused with mouse myeloma Sp2/0-Ag14 cells using polyethylene glycol (PEG). The culture supernatants were screened using enzyme-linked immunosorbent assay for binding to the pMAL-c2-hDGKδ-PA protein.

### 2.4 Western blot analyses

Lysates were boiled in sodium dodecyl sulfate sample buffer (Nacalai Tesque, Inc., Kyoto, Japan). The samples were electrophoresed using 5%–20% polyacrylamide gels under reducing condition (Nacalai Tesque, Inc.), and transferred onto a polyvinylidene difluoride (PVDF) membranes (Merck KGaA, Darmstadt, Germany). After blocking with 4% skim milk (Nacalai Tesque, Inc.) for 1 h, the membrane was incubated with DdMab-1 (1 μg/mL or 10 μg/mL) or NZ-1 (1 μg/mL) for 1 h, followed by incubation with HRP-conjugated anti-mouse immunoglobulins (1:2000 dilution; Agilent Technologies, Inc.) or HRP-conjugated anti-rat IgG (1:10,000 dilution; Sigma-Aldrich Corp.) for 1 h. The membrane was developed with the ImmunoStar LD Chemiluminescence Reagent (FUJIFILM Wako Pure Chemical Corporation) using the Sayaca-Imager (DRC Co., Ltd., Tokyo, Japan). All Western blot procedures were performed at room temperature.

## 3 Results

### 3.1 Establishment of anti-hDGKδ mAbs

**Fig. 1:**
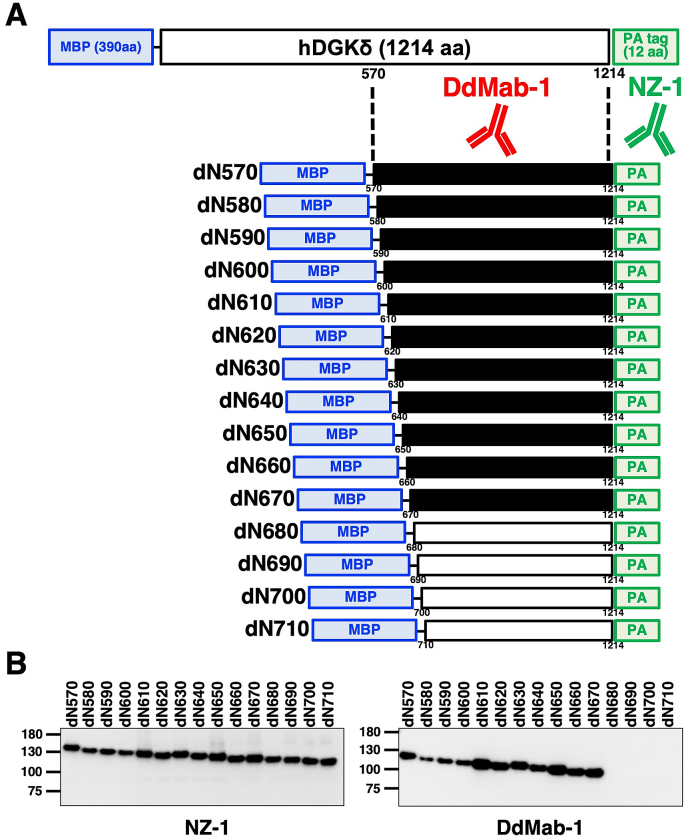
**Epitope mapping of DdMab-1 using deletion mutants of hDGKδ.** (A) Schematic illustration of DdMab-1 epitope mapping. Black bars, deletion mutants detected by DdMab-1. hDGKδ, human DGKδ; MBP, maltose-binding protein. (B) Cell lysates of hDGKδ N-terminal deletion mutants were electrophoresed, and then transferred onto a PVDF membrane. After blocking, the membrane was incubated with 1 μg/ml of DdMab-1 or anti-PA tag antibody (NZ-1).

Three B6D2F1/Slc mice were immunized by injecting 33 μg of the pMAL-c2-hDGKδ-PA protein into their footpad. Additional immunization with 50 μg of the pMAL-c2-hDGKδ-PA protein was performed via the tail base. The lymphocytes were fused with mouse myeloma Sp2/0-Ag14 cells using PEG. The culture supernatants were screened using enzyme-linked immunosorbent assay for the binding to the pMAL-c2-hDGKδ-PA protein. After Western blot screening, we established DdMab-1 (mouse IgG_2a_, kappa), which is useful for Western blot analysis against hDGKδ (Fig. 1).

### 3.2 Epitope mapping of DdMab-1 using deletion mutant of hDGKδ

Because DdMab-1 reacted with an N-terminal deletion mutant (dN570) of hDGKδ, we then produced an additional 14 N-terminal deletion mutants (dN580, dN590, dN600, dN610, dN620, dN630, dN640, dN650, dN660, dN670, dN680, dN690, dN700, and dN710), and performed western blotting to detect the location of the epitope (Fig. 1A). As shown in Fig. 1B, DdMab-1 recognized dN580, dN590, dN600, dN610, dN620, dN630, dN640, dN650, dN660, and dN670, but not dN680, dN690, dN700, and dN710 mutants. All of these deletion mutants were detected by the NZ-1 anti-PA tag mAb (Fig. 1B). Hence, the N-terminus of DdMab-1 epitope was found to be located between amino acids 670 and 680 of hDGKδ.

### 3.3 Epitope mapping of DdMab-1 using point mutants of hDGKδ

**Fig. 2:**
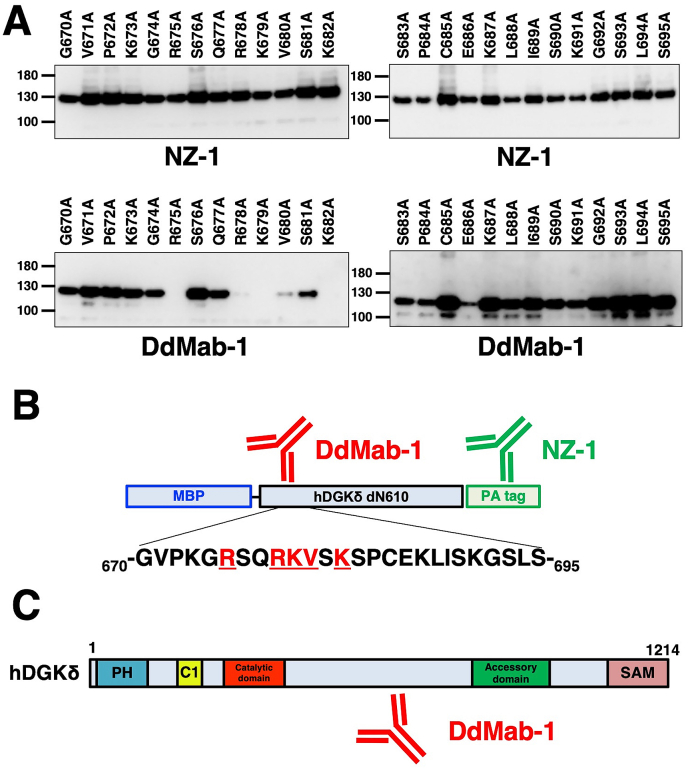
**Epitope mapping of DdMab-1 using point mutants of hDGKδ.** (A) Cell lysates of point mutants of dN610 were electrophoresed, and then transferred onto PVDF membranes. After blocking, the membranes were incubated with 10 μg/ml of DdMab-1 or 1 μg/ml of anti-PA tag antibody (NZ-1). (B) Schematic illustration of DdMab-1 epitope mapping. Underlined amino acids (Arg675, Arg678, Lys679, Val680 and Lys682) are important for binding of DdMab-1 to hDGKδ. (C) Schematic illustration of the hDGKδ structure. DdMab-1 epitope is located in between catalytic and accessory domains. PH, pleckstrin homology; SAM, sterile alpha motif.

We also produced further hDGKδ constructs including 26 alanine point mutations to identify the critical DdMab-1 epitope (G670A, V671A, P672A, K673A, G674A, R675A, S676A, Q677A, R678A, K679A, V680A, S681A, K682A, S683A, P684A, C685A, E686A, K687A, L688A, I689A, S690A, K691A, G692A, S693A, L694A, and S695A). All hDGKδ point mutants were recognized by NZ-1 (Fig. 2A). In contrast, DdMab-1 did not recognize R675A, R678A, K679A, and K682A mutants, and only weakly reacted with the V680A mutant (Fig. 2A), indicating that DdMab-1 binds to DGKδ via Arg675, Arg678, Lys679, Val680 and Lys682. These results are summarized in Fig. 2B. The identified DdMab-1 epitope of is located between the catalytic and accessory domains (Fig. 2C).

## 4 Discussion

Previously, we established DaMab-2 as an anti-DGKα mAb [22], DgMab-6 as an anti-DGKγ mAb [23], and DzMab-1 as an anti-DGKζ mAb [24] for immunocytochemistry. We further developed DaMab-8 as an anti-DGKα mAb [25] and DhMab-1 [26]/DhMab-4 [27] as anti-DGKη mAbs for immunohistochemistry. We determined their respective binding epitopes [[25], [26], [27], [28], [29], [30]]. Accordingly, DaMab-2 and DaMab-8 was found to bind to the Zn-finger and catalytic domains of DGKα, respectively [25,28]. DgMab-6 and DzMab-1 were shown to bind to the N-termini of DGKγ and DGKζ, respectively [29,30]. DhMab-1/DhMab-4 epitope was found to be located near the accessory domain of hDGKη [26,27]. These epitope analyses revealed that each sensitive and specific mAb for use in immunocytochemistry or immunohistochemistry against different DGK isotypes included different epitope regions.

Here, we reported a novel anti-hDGKδ mAb, DdMab-1, which is useful for Western blot analysis (Fig. 1, Fig. 2A). We also identified the binding epitope of DdMab-1 by western blotting, and found Arg675, Arg678, Lys679, Val680 and Lys682 to be important for DdMab-1 binding to hDGKδ. The epitope of DdMab-1 is located between catalytic and accessory domains (Fig. 2C). In our next study, we will investigate the utility of this mAb in immunocytochemistry and immunohistochemistry analyses for detection of hDGKδ protein in different tissues/organs including the stomach, testis, bone marrow, and lymph node.

## Funding

This research was supported in part by 10.13039/100009619AMED [grant numbers: JP20am0401013, JP20am0101078, JP20ae0101028] (Y.K.).

## References

[1] Topham M.K., Epand R.M. (2009). Mammalian diacylglycerol kinases: molecular interactions and biological functions of selected isoforms. Biochim. Biophys. Acta.

[2] Goto K., Hozumi Y., Nakano T., Saino S.S., Kondo H. (2007). Cell biology and pathophysiology of the diacylglycerol kinase family: morphological aspects in tissues and organs. Int. Rev. Cytol..

[3] Berridge M.J., Irvine R.F. (1984). Inositol trisphosphate, a novel second messenger in cellular signal transduction. Nature.

[4] Newton A.C. (1997). Regulation of protein kinase C. Curr. Opin. Cell Biol..

[5] Parekh D.B., Ziegler W., Parker P.J. (2000). Multiple pathways control protein kinase C phosphorylation. EMBO J..

[6] Mérida I., Arranz-Nicolás J., Rodríguez-Rodríguez C., Ávila-Flores A. (2019). Diacylglycerol kinase control of protein kinase C. Biochem. J..

[7] Nishizuka Y. (1984). The role of protein kinase C in cell surface signal transduction and tumour promotion. Nature.

[8] Fang Y., Vilella-Bach M., Bachmann R., Flanigan A., Chen J. (2001). Phosphatidic acid-mediated mitogenic activation of mTOR signaling. Science.

[9] English D., Cui Y., Siddiqui R.A. (1996). Messenger functions of phosphatidic acid. Chem. Phys. Lipids.

[10] Sakane F., Imai S., Kai M., Wada I., Kanoh H. (1996). Molecular cloning of a novel diacylglycerol kinase isozyme with a pleckstrin homology domain and a C-terminal tail similar to those of the EPH family of protein-tyrosine kinases. J. Biol. Chem..

[11] Fagerberg L., Hallström B.M., Oksvold P., Kampf C., Djureinovic D., Odeberg J., Habuka M., Tahmasebpoor S., Danielsson A., Edlund K., Asplund A., Sjöstedt E., Lundberg E., Szigyarto C.A., Skogs M., Takanen J.O., Berling H., Tegel H., Mulder J., Nilsson P., Schwenk J.M., Lindskog C., Danielsson F., Mardinoglu A., Sivertsson A., von Feilitzen K., Forsberg M., Zwahlen M., Olsson I., Navani S., Huss M., Nielsen J., Ponten F., Uhlén M. (2014). Analysis of the human tissue-specific expression by genome-wide integration of transcriptomics and antibody-based proteomics. Mol. Cell. Proteomics.

[12] Wang D.S., Shaw G. (1995). The association of the C-terminal region of beta I sigma II spectrin to brain membranes is mediated by a PH domain, does not require membrane proteins, and coincides with a inositol-1,4,5 triphosphate binding site. Biochem. Biophys. Res. Commun..

[13] Touhara K., Inglese J., Pitcher J.A., Shaw G., Lefkowitz R.J. (1994). Binding of G protein beta gamma-subunits to pleckstrin homology domains. J. Biol. Chem..

[14] Konishi H., Kuroda S., Kikkawa U. (1994). The pleckstrin homology domain of RAC protein kinase associates with the regulatory domain of protein kinase C zeta. Biochem. Biophys. Res. Commun..

[15] Stapleton D., Balan I., Pawson T., Sicheri F. (1999). The crystal structure of an Eph receptor SAM domain reveals a mechanism for modular dimerization. Nat. Struct. Biol..

[16] Buzovetsky O., Tang C., Knecht K.M., Antonucci J.M., Wu L., Ji X., Xiong Y. (2018). The SAM domain of mouse SAMHD1 is critical for its activation and regulation. Nat. Commun..

[17] Crotty T., Cai J., Sakane F., Taketomi A., Prescott S.M., Topham M.K. (2006). Diacylglycerol kinase delta regulates protein kinase C and epidermal growth factor receptor signaling. Proc. Natl. Acad. Sci. U. S. A..

[18] Cai J., Crotty T.M., Reichert E., Carraway K.L., Stafforini D.M., Topham M.K. (2010). Diacylglycerol kinase delta and protein kinase C(alpha) modulate epidermal growth factor receptor abundance and degradation through ubiquitin-specific protease 8. J. Biol. Chem..

[19] Chibalin A.V., Leng Y., Vieira E., Krook A., Björnholm M., Long Y.C., Kotova O., Zhong Z., Sakane F., Steiler T., Nylén C., Wang J., Laakso M., Topham M.K., Gilbert M., Wallberg-Henriksson H., Zierath J.R. (2008). Downregulation of diacylglycerol kinase delta contributes to hyperglycemia-induced insulin resistance. Cell.

[20] Fujii Y., Kaneko M., Neyazaki M., Nogi T., Kato Y., Takagi J. (2014). PA tag: a versatile protein tagging system using a super high affinity antibody against a dodecapeptide derived from human podoplanin. Protein Expr. Purif..

[21] Kato Y., Kaneko M.K., Kuno A., Uchiyama N., Amano K., Chiba Y., Hasegawa Y., Hirabayashi J., Narimatsu H., Mishima K., Osawa M. (2006). Inhibition of tumor cell-induced platelet aggregation using a novel anti-podoplanin antibody reacting with its platelet-aggregation-stimulating domain. Biochem. Biophys. Res. Commun..

[22] Nakano T., Ogasawara S., Tanaka T., Hozumi Y., Mizuno S., Satoh E., Sakane F., Okada N., Taketomi A., Honma R., Nakamura T., Saidoh N., Yanaka M., Itai S., Handa S., Chang Y.W., Yamada S., Kaneko M.K., Kato Y., Goto K. (2017). DaMab-2: anti-human DGKalpha monoclonal antibody for immunocytochemistry. Monoclon. Antibodies Immunodiagn. Immunother..

[23] Nakano T., Ogasawara S., Tanaka T., Hozumi Y., Yamaki A., Sakane F., Shirai Y., Nakamura T., Yanaka M., Yamada S., Kaneko M.K., Kato Y., Goto K. (2018). DgMab-6: antihuman DGKgamma monoclonal antibody for immunocytochemistry. Monoclon. Antibodies Immunodiagn. Immunother..

[24] Nakano T., Ogasawara S., Tanaka T., Hozumi Y., Sano M., Sayama Y., Yamada S., Kaneko M.K., Kato Y., Goto K. (2019). DzMab-1: anti-human DGKzeta monoclonal antibody for immunocytochemistry. Monoclon. Antibodies Immunodiagn. Immunother..

[25] Sano M., Kaneko M.K., Suzuki H., Kato Y. (2020). Establishment and epitope mapping of anti-diacylglycerol kinase alpha monoclonal antibody DaMab-8 for immunohistochemical analyses. Monoclon. Antibodies Immunodiagn. Immunother..

[26] Sano M., Kaneko M.K., Kato Y. (2020). Epitope mapping of DhMab-1: an antidiacylglycerol kinase monoclonal antibody monoclon. Antib. Immunodiagn. Immunother..

[27] Asano T., Sano M., Takei J., Sayama Y., Kaneko M.K., Kato Y. (2020). Epitope mapping of the anti-diacylglycerol kinase monoclonal antibody DhMab-4 for immunohistochemical analysis. Monoclon. Antibodies Immunodiagn. Immunother..

[28] Sano M., Kaneko M.K., Kato Y. (2019). Epitope mapping of antidiacylglycerol kinase alpha monoclonal antibody DaMab-2. Monoclon. Antibodies Immunodiagn. Immunother..

[29] Sano M., Kaneko M.K., Kato Y. (2019). Epitope mapping of antihuman diacylglycerol kinase gamma monoclonal antibody DgMab-6. Monoclon. Antibodies Immunodiagn. Immunother..

[30] Sano M., Kaneko M.K., Kato Y. (2019). Epitope mapping of anti-diacylglycerol kinase zeta monoclonal antibody DzMab-1 for immunohistochemical analyses. Monoclon. Antibodies Immunodiagn. Immunother..

